# Natural Compounds With Antibacterial Activity Against *Cronobacter* spp. in Powdered Infant Formula: A Review

**DOI:** 10.3389/fnut.2020.595964

**Published:** 2020-11-23

**Authors:** Gökçe Polat Yemiş, Pascal Delaquis

**Affiliations:** ^1^Department of Food Engineering, Sakarya University, Serdivan, Turkey; ^2^Summerland Research and Development Research Centre, Agriculture and AgriFood Canada, Summerland, BC, Canada

**Keywords:** natural, antimicrobials, *Cronobacter*, safety, powdered infant formula

## Abstract

Bacteria from the genus *Cronobacter* are opportunistic foodborne pathogens capable of causing severe infections in neonates, the elderly and immunocompromised adults. The majority of neonatal infections have been linked epidemiologically to dehydrated powdered infant formulas (PIFs), the majority of which are manufactured using processes that do not ensure commercial sterility. Unfortunately, the osmotolerance, desiccation resistance, mild thermotolerance and wide-ranging minimum, optimum and maximum growth temperatures of *Cronobacter* spp. are conducive to survival and/or growth during the processing, reconstitution and storage of reconstituted PIFs. Consequently, considerable research has been directed at the development of alternative strategies for the control of *Cronobacter* spp. in PIFs, including approaches that employ antimicrobial compounds derived from natural sources. The latter include a range of phytochemicals ranging from crude extracts or essential oils derived from various plants (e.g., thyme, cinnamon, clove, marjoram, cumin, mint, fennel), to complex polyphenolic extracts (e.g., muscadine seed, pomegranate peel, olive oil, and cocoa powder extracts), purified simple phenolic compounds (e.g., carvacrol, citral, thymol, eugenol, diacetyl, vanillin, cinnamic acid, trans-cinnamaldehyde, ferulic acid), and medium chain fatty acids (monocaprylin, caprylic acid). Antimicrobials derived from microbial sources (e.g., nisin, other antibacterial peptides, organic acids, coenzyme Q_0_) and animal sources (e.g., chitosan, lactoferrin, antibacterial peptides from milk) have also been shown to exhibit antibacterial activity against the species. The selection of antimicrobials for the control of *Cronobacter* spp. requires an understanding of activity at different temperatures, knowledge about their mode of action, and careful consideration for toxicological and nutritional effects on neonates. Consequently, the purpose of the present review is to provide a comprehensive summary of currently available data pertaining to the antibacterial effects of natural antimicrobial compounds against *Cronobacter* spp. with a view to provide information needed to inform the selection of compounds suitable for control of the pathogen during the manufacture or preparation of PIFs by end users.

## Introduction

Fatal bacterial infections in neonates caused by “yellow-pigmented coliforms” were first reported in the early 1960s (1). Early clinical isolates were classified as strains of *Enterobacter cloacae* until comparative analysis by DNA–DNA hybridization showed they belong to a distinct species that was initially named *Enterobacter sakazakii* (2). Further genomic analysis by ribotyping, amplified fragment length polymorphism and 16S rDNA sequencing eventually provided evidence to support assignment of *E. sakazakii* and other closely related *Enterobacter* species to the novel genus *Cronobacter* (3). The List of Prokaryotic names with Standing in Nomenclature (LPSN) presently includes seven species of *Cronobacter* with a validly published and correct name, including *Cronobacter sakazakii, Cronobacter malonaticus, Cronobacter universalis, Cronobacter turicensis, Cronobacter muytjensii, Cronobacter dublinensis* and *Cronobacter condimenti* (https://www.bacterio.net/genus/cronobacter). *C. sakazakii* is the most frequently reported clinical isolate and is considered to be the prototype species for the genus (4, 5). However, all *Cronobacter* spp. with the exception of *C. condimenti* have been recovered from clinical specimens (6). *C. sakazakii* and *C. malonaticus* are currently the major species of public health concern, followed by *C. turicensis, C. universalis, C. muytjensii*, and *C. dublinensis* (5). *Cronobacter* infections typically affect hosts with immature or compromised immune systems, primarily neonates and infants, and to a lesser extent the elderly or individuals from all age groups with underlying chronic disease. Consequently, *Cronobacter* spp. are considered opportunistic human pathogens. Infections in neonates can lead to septicemia, necrotizing enterocolitis or severe meningitis with estimated case-fatality rates of 10, 20, and 42%, respectively, and to severe neurological sequelae upon recovery (7–9). Symptoms of infection in adults include wound and urinary tract infections, gastroenteritis, appendicitis, conjunctivitis, biliary sepsis, pneumonia, septicemia, and osteomyelitis (10). *Cronobacter* infections were considered exceptional and sporadic occurrences until the late 1980s when several clusters were reported in neonatal care units (11). Clinical investigation of a landmark incident in a US hospital showed that neonates were infected by enteral administration of reconstituted powdered infant formula (PIF) (12). Epidemiological investigations of similar incidents in other jurisdictions have confirmed that PIFs can serve as a vehicle for the foodborne transmission of *C. sakazakii* to neonates (13–15). Only one suspected foodborne outbreak involving ostensibly healthy and older individuals has been reported to date. Yong et al. (16) presented evidence that food contaminated with *Cronobacter* spp. consumed in a senior high school canteen led to an outbreak of acute gastroenteritis that resulted in 124 suspected, 12 probable, and 20 confirmed cases. Molecular analysis of isolates recovered from clinical, food, or environmental samples revealed the presence of both *C. sakazakii* (four isolates from two sequence types determined by multilocus sequence typing) and *C. malonaticus* (two isolates from one sequence type). However, the whole genome sequences of two *C. sakazakii* isolates recovered from a food sample and a clinical specimen differed by only five single nucleotide polymorphisms, which was highly suggestive of an epidemiological link.

While *Cronobacter* infections remain uncommon, alarmingly high case-fatality rates in neonates and uncertainty about transmission outside hospital care settings have prompted considerable research to determine the origin, distribution and fate of this emerging foodborne pathogen in food chains. Despite these efforts, the primary habitats of *Cronobacter* spp. remain unknown. Infrequent isolation from livestock and limited survival in the animal gut are indicative of a non-zoonotic nature, although contamination of meat and milk have been reported (17–19). A review and meta-analysis of data published between 2008–14 revealed a prevalence of 5.7% in meat products and 19.0% in plant based foods or food ingredients, which is suggestive of a stronger association with plants or environments in which they are grown (20). Irrespective of their primary habitat, *Cronobacter* spp. have been isolated from diverse dehydrated food products (PIFs, infant cereals, dairy-based preparations, flours, pasta, candies, spices, herbs, and nuts), fresh or frozen vegetables, and both natural (soil, water, insects) and man-made (hospitals, households, food storage and processing facilities) environments (20–23).

Current understanding about the fate of *Cronobacter* spp. in food systems is primarily derived from research concerned with the role of PIFs in foodborne transmission. PIFs intended to serve as complete or partial substitutes for human milk at birth or after the introduction of solid food (follow-up formulas) contain mixtures of protein, fat, carbohydrates, vitamins, minerals, and other functional ingredients (e.g., essential fatty acids, nucleotides) in proportions needed to achieve nutrient contents mandated by national or international regulatory standards. Intact bovine milk powder is the most common source of protein, although specialized formulas containing hydrolyzed casein or proteins derived from plant sources such as soy bean are used for feeding of neonates with underlying pathologies or to accommodate cultural or religious practices (24). Manufacture of PIF products is accomplished by spray-drying of the mixed ingredients solubilized in water (wet processes), by mixing of heat-labile ingredients with a previously spray-dried base powder (dry processes), or by a combination of both approaches (25). Despite the application of heat at one or more stages of these processes PIF is not a sterile food; *Cronobacter* spp. are routinely detected in microbiological analysis of commercial products (26, 27). For example, a recent survey of 128 products in Latin American markets revealed a prevalence rate of 4.7% (28). Microbiological assessments of milk powder and PIF manufacturing environments and processes have shown that *Cronobacter* spp. may derive from extrinsic sources, notably dry ingredients, or intrinsically contaminated sites where specific strains may persist over long periods of time (29–33). Moreover, most strains examined to date have shown higher resistance to potentially lethal osmotic and dessication stresses than other human pathogen belonging to the family Enterobacteriaceae. Dessication resistance likely contributes to the environmental persistence of *Cronobacter* spp. in some niches within manufacturing plants, and to long term survival in powdered milk and PIF (34–36).

The suspected role of PIF in the transmission of infections has prompted examination of *Cronobacter* behavior during reconstitution in water and subsequent handling, including storage for later use. Incipient work by Nazarowec-White and Farber (37) showed that *C. sakazakii* could survive reconstitution with water heated to 52–60°C. Data from additional studies conducted with numerous strains over a wider range of temperatures and in different substrates suggests that most *Cronobacter* spp. are mildly thermotolerant, although strain-associated stress tolerance or prior heat adaptation can enhance thermal stability (38–41). In response to the risk implied by latent contamination of PIFs with infectious bacteria the WHO recommends reconstitution in water at a minimum temperature of 70°C, conditions which have been shown to reduce *C. sakazakii* by >5 log_10_ cycles (42, 43). Reconstitution at ≥70°C is often impractical, however, as high temperatures can lead to curdling or other undesirable organoleptic changes, cause depletion of heat sensitive nutrients, and introduce scald or burn hazards particularly in home settings (27, 44). Consequently, lower temperatures are endorsed in some jurisdictions despite experimental evidence of limited thermal inactivation at lower temperatures. Moreover, growth of *C. sakazakii* has been reported to occur in reconstituted PIF stored between 5.5 and 47°C, conditions that can occur when feeding is delayed or during storage (15, 21, 41). Additionally, recent work has shown that *C. sakazakii* is readily transferred from caregiver hands and utensils to reconstituted PIF, thereby highlighting the significance of contact surfaces as reservoirs of contamination (45). Adherence to and biofilm formation have been demonstrated on a wide range of materials used in the manufacture of equipment, tools and utensils used in hospital and home settings (46–48). Biofilm formation also contributes to the persistence of *Cronobacter* on surfaces by enhancing resistance to adverse environmental stresses, including chemicals used in cleaning and sanitation of food processing facilities and equipment (49).

The risk of contamination with *Cronobacter* spp. is an enduring food safety challenge for the PIF industry, public health authorities and consumers worldwide. Manufacturers have adopted risk mitigation strategies that primarily rely on rigorous microbiological analysis of raw materials, improved cleaning and sanitation of the manufacturing environment, and enhanced testing of finished products. Despite these efforts, levels of contamination detected through recent surveys clearly show that the risk persists (28). Because the modification of existing industrial processes is constrained by the heat lability of PIF ingredients, alternative non-thermal physical treatments meant to inactivate microbial contaminants without affecting ingredient stability have been investigated or are under study (reviewed in (50, 51)). To date, none have been adapted to the production of PIF on an commercial scale. The use of synthetic preservatives is likewise impractical due to regulatory restrictions and enduring concerns about the negative effects of man-made food additives on human health. Accordingly, natural antibacterial compounds (NACs) derived from plant, microbial or animal sources are under consideration as alternatives to synthetic chemical preservatives for the control of *Cronobacter* spp. in PIF. This approach is aligned with increasing consumer willingness to accept food additives and preservatives of natural origin over synthetic products (52). The present work is intended to provide a summary of current knowledge about NACs with antibacterial activity against *Cronobacter* spp., with a view to inform their application in the development of improved PIF manufacturing processes or the formulation of safer products. For example, NACs that increase the lethality of mild heat may find value in the development of alternative processes to enhance bacterial inactivation in manufacture or during reconstitution by end-users (53). Where possible, the mode of action, toxicological data, regulatory status and potential health benefits of specific NACs are provided.

## NACs From Plants With Antibacterial Activity Against *Cronobacter* Spp.

The scope of research on plants as sources of natural antimicrobials and their applications in food preservation or safety has expanded significantly in recent years (54). Plant components and extractives thereof are attractive alternative food additives because many have a long history of use, are likely to have received regulatory consent or benefit from the increasing availability of toxicological data needed to support requests for approval (55). Recognition of PIF as a vector for foodborne transmission has led to the assessment of numerous crude extracts, essential oils recovered by distillation of whole plants or their parts, and purified phytochemicals for the control of *Cronobacter* spp. (Table 1). Crude extracts obtained in water or an alcoholic solvent are generally not modified after evaporation of the liquid phase and contain several bioactive constituents. For example, a tea extract examined by Li et al. (61) was reported to contain catechins, flavonoids, phenolic acids, anthocyanins, malic acid, and citric acid. The latter is typical of crude plant extracts which tend to consist of complex mixtures of compounds from different classes, notably large and diverse fractions of phenolic compounds referred to collectively as polyphenolics. Most crude extracts listed in Table 1 contain polyphenolics known to have antibacterial properties, although additive or synergistic effects due to co-extracted plant constituents, notably organic acids, likely contribute to overall antibacterial activity (57). The mode of action of crude extracts is accordingly complex, however damage to the bacterial membrane leading to loss of function and cellular integrity induced by polyphenolics contributes significantly to overall antibacterial effects (83). Two extracts (cocoa powder and polyphenolic tea extract) were shown to exert bacteriostatic effects and reduce the growth of *Cronobacter* spp. in reconstituted PIF. Crude plant extracts are economically attractive food additives due to their low cost of production in comparison purified phytochemicals. However, variability in antibacterial activity due to differences in composition resulting from varietal, agronomic or production factors tend to limit practical applications in foods. Concentrations of compounds with antibacterial activity may also be lower than those of co-extracted compounds that can lend undesirable physico-chemical or organoleptic properties to target food products. Similar constraints hamper food applications for essential oils, complex mixtures of volatile lipophilic terpenoids, phenylpropanoids, or short-chain aliphatic hydrocarbon derivatives (84). Results from four scientific reports listed in Table 1 show that essential oils recovered from several aromatic plant species show antibacterial activity against *Cronobacter* spp. Moreover, Al-Nabulsi et al. (65) found that growth of *C. sakazakii* in reconstituted PIF was inhibited by supplementation with cinnamon or fir essential oils and that mixtures of the two were bactericidal, reducing populations by >6 log_10_ after 3 h of incubation at 37°C. These results support the widely held view that the activity of essential oils stems from additive or synergistic antimicrobial effects involving multiple components, and underscores the need to determine concentrations of key active compounds needed to ensure consistent activity in foods (85).

**Table 1:** Plant-derived crude extracts, essential oils, and purified phytochemicals with antibacterial activity against *Cronobacter* spp.

	**Agents with *in vitro* antibacterial activity**	**Composition**	**Demonstrated or suggested mode of action**	**Antibacterial activity in PIF**	**References**
Crude extracts	Aqueous extracts of red muscadine seed; muscadine juice	Malic, tartaric, tannic acids; polyphenols (gallic acid, catechin, epicatechin, ellagic acid, resveratrol)	Not determined. Suggested synergy between components	Not determined	(56, 57)
	Cocoa powder	Polyphenol rich	Not determined	Bacteriostatic in reconstituted PIF incubated at 37°C	(58)
	Proanthocyanidin- rich methanolic blueberry extract; blueberry juice	Not determined	Loss of membrane integrity, altered fatty acid profile, disruption of metabolism	Not determined	(59)
	Methanolic extracts of black pepper and cinnamon bark	Not determined	Inhibition of quorum sensing and biofilm formation at sub-inhibitory concentrations	Not determined	(60)
	Polyphenolic tea extract	Not determined	Membrane damage leading to cytoplasmic leakage; pH effects	Bacteriostatic effect in reconstituted PIF incubated at 37°C; enhanced at pH ≤ 4	(61)
	Polyphenolic olive oil extract	Hydroxytyrosol, tyrosol, phenolic acids	Reduction of intracellular ATP, membrane depolarization, decreased protein synthesis	Not determined	(62)
	Polyphenolic rich pomegranate peel extract	Elligitannins, α, β-punicalagin, ellagic acid and derivatives, punicalin	Not determined	Not determined	(63)
Essential oils	Cinnamon, clove, lemongrass, laurel, oregano essential oils	Not Determined	Not determined	Not determined	(64)
	Cinnamon, fir essential oils	Nor determined	Not determined. Suggested synergy between phenolic compounds, organic acids and other components	Bacteriostatic effects in reconstituted PIF incubated at 37°C with fir and cinnamon oils; bactericidal effects with fir + cinnamon oil	(65)
	Thyme, clove, ginger extracts obtained by hydrodistillation	Not determined	Not determined	Not determined	(66)
	Thyme, cinnamon, marjoram essential oils	Not determined	Not determined. Suggested synergy between phenolic compounds	Not determined	(67)
Purified phytochemicals	Carvacrol, thymol, eugenol, diacetyl, cinnamic acid	NA	Not determined	Not determined	(68)
	Trans-cinnamaldehyde	NA	No determined. Suggested disruption of bacterial cell membrane	Bactericidal effects in reconstituted PIF incubated at 37 and 8°C; Reduced resistance to acid and heat in reconstituted PIF	(69, 70)
	Vanillin, vanillic acid	NA	Disruption of bacterial cell membrane	Reduced heat resistance during reconstitution; Bactericidal effects in reconstituted PIF incubated at 21 and 10°C	(71, 72)
	Caprylic acid	NA	Disruption of bacterial cell membrane; synergistic effects when used in combination with citric acid or vanillin	Reduced heat resistance during reconstitution; Bacteriostatic effects in reconstituted PIF incubated at 40°C	(73, 74)
	Citral	NA	Reduction of intracellular ATP, cell membrane hyperpolarization, reduction in cytoplasmic pH.	Not determined	(75)
	Syringic acid	NA	Reduction of intracellular ATP, cell membrane hyperpolarization, reduction in cytoplasmic pH.	Not determined	(76)
	Ferulic acid	NA	Disruption of bacterial cell membrane	Not determined	(77)
	Thymoquinone	NA	Disruption of bacterial cell membrane; Reduced stress tolerance; Decreased motility, quorum sensing, endotoxin production, biofilm formation at sub-inhibitory concentrations	Reduced resistance to heat during reconstitution; Reduced resistance to acid, heat, osmotic stress in reconstituted PIF	(78–80)
	Carvacrol + citral	NA	Disruption of energy maintenance, membrane repair and proton motive force at sub-inhibitory concentrations	Not determined	(81)
	Coenzyme Q_0_	NA	Reduction of intracellular ATP; Disruption of bacterial cell membrane; inhibition of biofilm formation	Reduced resistance to heat during PIF reconstitution;	(82)

Extraction and purification of NACs from plants circumvents challenges occasioned by variable crude extract or essential oil composition. Purified preparations of the phytochemicals listed in Table 1 are readily available from commercial sources. Most are known to exert antimicrobial effects primarily by disruption of the bacterial cell membrane. Trans-cinnamaldehyde, vanillin, ethyl vanillin, vanillic acid, thymoquinone, and Coenzyme Q_0_ have been shown to reduce the thermotolerance of *Cronobacter* spp. during reconstitution and to provide antibacterial effects during subsequent storage. Overall, the reported nature and magnitude of measured effects vary with type of compound, level of PIF supplementation and temperature. For example, Amalaradjou et al. (69) reported that trans-cinnamaldehyde exerts time- and temperature-dependent bactericidal activity against *C. sakazakii* in reconstituted PIF during storage at 4, 8, 23, and 37°C. In contrast, supplementation with caprylic acid could only elicit bacteriostatic effects against the same species in reconstituted PIF stored at 40°C (74). The effects of these compounds on the thermotolerance of *Cronobacter* are more consistent. Amalaradjou et al. (70) showed that complete thermal inactivation of *C. sakazakii* could be achieved by heating reconstituted PIF supplemented with 70 μM trans-cinnamaldehyde at 60°C for 10 min, but that longer heating times were required to achieved the same effect at lower supplementation levels or temperatures. Likewise, *C. sakazakii* was completely inactivated by heating reconstituted PIF supplemented with 30 mmol l^−1^ thymoquinone at 55°C for 10 min, and longer treatment times were needed to achieved the same effect at lower concentrations or temperatures (78). Caprylic acid (73), coenzyme Q_0_ (82), vanillin, ethyl vanillin, and vanillic acid (72) have also been found to lower the thermotolerance of *C. sakazakii* during reconstitution. Moreover, the bactericidal activity of vanillin, ethyl vanillin, and vanillic acid were sustained during subsequent storage at 21 and 5°C, which illustrates that some phytochemical compounds can contribute antibacterial effects at multiple stages during the manufacture or preparation of PIF by end-users.

The volatility, susceptibility to conversion and degradation reactions, intense organoleptic character and poor solubility of many plant extracts, essentials oils or phytochemicals have long hindered wider use in food preservation. However, considerable progress has been achieved in the development of novel technologies for the delivery of food additives that are intended to overcome these limitations, notably encapsulation methods that stabilize active components and enable their release of over variable time periods, at specific temperatures or at different sites within food matrices (reviewed in (86)). To date, the merit of these technologies for the delivery of NACs for the control of *Cronobacter* in PIF remains unexplored. Encapsulation platforms that provide controlled release at temperatures applied during the manufacture, preparation or storage of reconstituted PIF could provide the means to ensure delivery when contaminants are most vulnerable to their effects or when the risk of proliferation is highest.

## NACs From Microbial Sources With Antibacterial Activity Against *Cronobacter* Spp.

Several NACs from microbial sources and their antibacterial activity against *Cronobacter* spp. are shown in Table 2. Probiotics, live microorganisms which when administered in adequate amounts confer a health benefit on the host, are presently used in some countries for pediatric care. Commercial PIFs products containing live preparations of *Lactobacillus* or *Bifidobacterium* spp. are available in the marketplace but the effect of probiotic supplementation on the behavior of *Cronobacter* spp. during or after reconstitution is unknown. The use probiotic bacteria for the explicit control of *Cronobacter* spp. infections was investigated by Collado et al. (93) who showed that species of *Lactobacillus, Bifidobacterium* and *Streptococcus* competitively excluded, inhibited and displaced *C. sakazakii* in a human intestinal model system. Despite evidence of their antibacterial properties, there have been few additional attempts to exploit the use of live probiotic bacteria for the control of human pathogens in PIF, likely in response to on-going controversy about the efficacy, safety, variability, quality, labeling, and lack of standards for the use of probiotic products destined for neonatal care (94, 95). NACs derived from microbial sources continue to attract interest, however, notably whole inactivated cells or crude cell extracts that retain bioactivity, which have been termed “parabiotics” (96), or purified soluble factors (products or metabolic byproducts) secreted by live microorganisms or released after lysis, which have been described as “postbiotics” (97). Hayes et al. (87) showed that addition of a crude cell-free extract prepared from a caseinate medium fermented by the common probiotic bacterium *Lactobacillus acidophilus* could inhibit *C. sakazakii* in reconstituted PIF. Antimicrobial effects were attributed to antimicrobial peptides (caseicin A & B) derived from the degradation of bovine casein by microbial enzymes. Charchoghlyan et al. (89) found that a purified heat inactivated aqueous extract of skim milk fermented with a commercial probiotic strain of *Lactobacillus acidophilus* used to supplement PIF also inactivated *C. sakazakii* in the reconstituted product stored at 37°C. The composition of the extract was not provided by the authors of the study, who offered that acids released during fermentation were likely responsible for antibacterial effects. A similar conclusion was reached by Kim et al. (90) in reference to the mechanism responsible for inhibition of *C. sakazakii* by cell free supernatants of laboratory grown cultures of *Lactobacillus kefiri* and *Lactobacillus kefiranofaciens* isolated from kefir, and a commercial probiotic strain of *Bifidobacterium longum*. It must be noted here that current international (e.g., CODEX STAN 72-1981) or national standards for ingredients or additives permissible in PIF do not include D-lactic acid, a metabolic by-product released during fermentation by *L. acidophilus, L., kefiri*, and *L. kefiranofaciens*. Probiotic bacteria such as *Bifidobacterium longum* that do not produce D-lactic acid could be used to circumvent the problem. However, uncertainty concerning the composition and variability in bioactive components in crude cell-free extracts derived from any microbial species is a barrier to broader food applications. The problem can be avoided by the isolation, purification and characterization of antimicrobial compounds that accumulate in growth media. For example, the examination of cell-free supernatants from laboratory grown cultures of *L. acidophilus* and *L. casei* by Awaisheh et al. (88) revealed that both species produce heat-labile bacteriocins, proteinaceous or peptidic molecules with antibacterial activity against *C. sakazakii*. To date, nisin is the only antibacterial peptide to receive close scrutiny for enhancement of PIF safety. Nisin, a polycyclic peptide bacteriocin produced by *Lactococcus lactis*, is presently permitted as a food additive in over 50 countries, primarily for the extension of shelf-life or the prevention of quality defects in dairy products (98). The antibacterial activity of nisin is mainly due to depolarizing effects and consequent disruption of the cytoplasmic cell membrane. Gram-positive bacteria are more resistant to this effect than Gram-negative bacteria in which the cell membrane is surrounded by a lipopolysaccharide outer membrane. Consequently, means to destabilize and permeabilize the outer membrane by physical (mild heat, sonication) or chemical means (metal chelators, EDTA, disodium pyrophosphate, sodium hydrogen orthophosphate, citric acid, lactic acid) are often used in conjunction with nisin to improve antibacterial activity against Gram-negative bacteria such as *Cronobacter* spp. Al-Nabulsi et al. (99) found that reconstitution of PIF at 55°C did not significantly improve the antibacterial activity of nisin against 5 *Cronobacter* strains. In contrast, Campion et al. (92) observed strong bactericidal activity against *C. sakazakii* in reconstituted PIF supplemented with a commercial nisin preparation (Nisaplin) and citric acid, a food additive permitted in infant formula. This finding suggests that antibacterial strategies based on synergistic effects between nisin and additives compliant with regulatory standards for PIF merit further investigation. Corresponding efforts should be directed at the assessment of alternative sources of antimicrobial peptides, including other commercial bacteriocin products or the array of peptides derived from probiotics or bacterial starter cultures described in the scientific literature (100).

**Table 2:** Natural antimicrobials from microbial sources with antibacterial activity against *Cronobacter* spp.

**Antibacterial activity *in vitro***	**Demonstrated or suggested mode of action**	**Antibacterial activity in PIF**	**References**
Cell-free extract of *Lactobacillus acidophilus* grown in caseinate, containing antimicrobial peptides caseicin A & B	Not determined	Bactericidal effect in reconstituted PIF incubated at 37 and 6°C	(87)
Heat-labile bacteriocins in cell-free extracts of *Lactobacillus casei* and *L. acidophilus*	Not determined	Bacteriostatic effect in reconstituted PIF incubated at 37°C	(88)
Cell-free extract of *Lactobacillus acidophilus*	Not determined. Suggested effect of organic acids released by *Lactobacillus acidophilus* during growth	Bactericidal effect in reconstituted PIF incubated at 37°C	(89)
Cell-free extracts of *Lactobacillus kefiri*	Disruption of bacterial cell membrane	Not determined	(90)
Cell-free extracts of *Lactobacillus acidophilus, L. bulgaricus L. casei rhamnosus, L. paracasei L. salivarius*	Disruption of bacterial cell membrane	Not determined	(91)
Nisin +citric acid	Not determined	Bactericidal effect in reconstituted PIF incubated at room temperature when mixed with citric acid	(92)

## NACs From Animal Sources With Antibacterial Activity Against *Cronobacter* Spp.

Selected NACs from animal sources and their antibacterial activity against *Cronobacter* spp. are shown in Table 3. The first natural antimicrobial derived from animal sources considered for the control of *Cronobacter* spp. in PIF was lactoperoxidase, an enzyme that occurs in milk, colostrum, tears, saliva, and other mammalian secretions (101). Lactoperoxidase catalyzes the oxidation of thiocyanate to hypothiocyanous acid and hypothiocyanate by H_2_O_2_ and generates intermediate products with antimicrobial properties. The “lactoperoxidase (LPO) system” relies on the interaction of all three components which must be present in sufficient amounts to initiate the reaction. Gurtler and Beuchat (101) showed that addition of bovine LPO to reconstituted milk-based PIF could inhibit the growth of *C. sakazakii* at temperatures >21°C. However, use of the LPO system in PIF is hindered by the need for addition of an exogenous source of thiocyanate, a known goitrogen (106). Another antibacterial protein also found in mammalian secretory fluids, lactoferrin, is not bound by this restriction. Lactoferrin, a small glycoprotein, inhibits bacteria indirectly by the sequestration of iron from the environment and through direct antibacterial effects resulting from disruption of the outer Gram-negative bacterial membrane, leading to alterations in cell permeability and loss of viability (107). Indirect antibacterial effects mediated by alteration of host innate immune functions have also been reported (108). Bovine milk is the most common source of lactoferrin and several manufacturers provide purified preparations for use in pharmaceutical, cosmetic and food applications, including the supplementation of PIF. Usage of lactoferrin supplemented PIF is common in some countries for the prevention of neonatal sepsis and necrotizing enterocolitis despite continued uncertainty about efficacy (109). Experimentation *in vitro* has shown that *Cronobacter* spp. are highly susceptible to the direct antibacterial effects of lactoferrin (99). However, the same authors found no evidence of antibacterial effects in reconstituted PIF during storage at 10, 21, or 37°C, a result ascribed to interactions with food components that reduced the activity of lactoferrin, notably the divalent cations Ca^2+^, Mg^2+^, and Fe^3+^. Harouna et al. (102) attempted to improve the activity of lactoferrin by saturation with iron cations but the saturated form of the protein had no measurable antibacterial effect against *C. sakzakii*. Numerous novel antimicrobial peptides with enhanced antimicrobial activity have been synthesized through chemical or enzymatic hydrolysis of lactoferrin (110). Harouna et al. (103) prepared lactoferrin hydrolysates using pepsin, chymosin and microbial rennet that exhibited enhanced antibacterial activity against *C. sakazakii* in a microbiological medium. None were effective in reconstituted PIF at 37°C, however, which provided further evidence that PIF ingredients interfere with the activity of proteic or peptidic antimicrobials. The majority of NACs from animal sources considered for the enhancement of PIF safety have been derived from bovine milk. Antimicrobial peptides have been detected in the milk of other food animal species including sheep and goats (111). A recent report that growth of *C. sakazakii* is inhibited by strong, inherent antibacterial factors in camel milk suggested they are likely derived from the protein component (105). A database assembled by Wang et al. (112) lists 1,972 known antimicrobial peptides from animal sources, in addition to 321 from plants and many from fungi, protists or other life forms. One antimicrobial peptide from a non-bovine source, bicarinalin from ants, exhibited stronger bactericidal effects against *C. sakazakii* than ampicillin and tetracycline (104). Clearly, animals are a rich and largely untapped source of NACs that could find value in the enhancement of PIF safety.

**Table 3:** Natural antimicrobials from animal sources with antibacterial activity against *Cronobacter* spp.

**Antibacterial activity *in vitro***	**Demonstrated or suggested mode of action**	**Antibacterial activity in PIF**	**References**
Lactoperoxidase	Not determined; Suggested result of oxidation of sulfhydryl groups on enzymes and proteins in cytoplasmic membranes.	Bactericidal effect in reconstituted PIF incubated at 21, 30, 37°C	(101)
Bovine lactoferrin	Not determined	Limited bacteriostatic effect when high concentrations were added to reconstituted PIF stored at 21°C; no effect at 10°C	(99)
Iron-saturated bovine lactoferrin	Not determined. Suggested destabilization of bacterial membrane	Not determined; Limited bacteriostatic activity in whey	(102)
Cationic peptides from enzymatic hydrolysis of lactoferrin	Not determined	Limited bacteriostatic effects in combination with native lactoferrin	(103)
Bicarinalin (cationic peptide from ants)	Disruption of bacterial cell membrane	Not determined	(104)
Camel milk	Not determined	Not determined	(105)

## Nutritional, Toxicological, and Regulatory Considerations

Commercial PIF products are subject to regulations and regulatory oversight administered by national governments. All are based on the Codex Alimentarius “Standard for Infant Formula and Formulas for Special Medical Purposes Intended for Infants (CODEX STAN 72-1981),” which provides recommendations regarding the essential composition, nutritional quality and additives in formula. CODEX recommendations are adopted by national governments in various ways, but there is general consistency in the application of compositional and nutritional quality standards for PIF across the globe. In contrast, some disparities exist with respect to the use of additives due to variable legislative, legal or regulatory frameworks within different jurisdictions. In the United States, food ingredients are subject to provisions in the Code of Federal Regulations. Ingredients that are not listed in the Code can obtain Generally Recognized as Safe (GRAS) designation through a notification program which requires petitioners to provide historical and scientific evidence that a substance added to food is considered safe and suitable for exemption from the food additive tolerance requirements of the United States Food and Drug Administration. The GRAS status of selected NACs with antibacterial activity against *Cronobacter* spp. in PIF is shown in Table 4, along with health benefits that have been ascribed to them and pertinent toxicological data. The authors recognize that lack of knowledge about the metabolism of food additives old or new, difficulties in determining accurate levels of exposure, and susceptibility to toxicity have historically hindered safety assessments in the neonatal context (150). Likewise, the purported health benefits noted in Table 4 are largely derived from animal studies or human feeding trials conducted with children or adults, and extrapolation of results to the neonatal situation is challenging.

**Table 4:** Suggested health-promoting effects, toxicological data, safety assessments and regulatory status of selected natural antimicrobial compounds with antibacterial activity against *Cronobacter* spp. in PIF.

**NAC**	**Reported Health Benefits**	**Toxicology/safety assessments**	**Regulatory status**
Cocoa powder	Prevention of cardiovascular disease; improved blood pressure regulation, insulin resistance and vascular function; increased production of nitric oxide (NO) and antioxidant effects including delayed oxidation of low-density lipoprotein cholesterol, inhibition of ultraviolet-induced DNA oxidation (113).	Chronic dietary exposure not carcinogenic to rats (114); No evidence of toxic effects on the heart, liver, kidney, lungs, testis, and spleen of rats fed high oral doses (115).	Food ingredient.
Polyphenolic tea extracts	Black teas: prevention of cancer; obesity, antioxidant protective and anti-hyperglycemic effects (116). Green teas: prevention of cancer, obesity, metabolic syndrome, type 2 diabetes, cardiovascular diseases (117).	Suspected cytotoxicity of epigallocatechin 3-gallate, the major catechin present in green tea, in adults and children (117, 118).	Generally Recognized as Safe (GRAS) according to US Code of Federal Regulations (USCFR), Title 21, § 182.20, Essential oils, oleoresins (solvent-free), and natural extractives (including distillates (119). Listed as dietary supplements under the Health and Education Act of 1994 (120).
Cinnamon essential oil, trans-cinnamaldehyde	Antitumour, anti-inflammatory and analgesic, anti-diabetic and anti-obesity, antibacterial and antiviral, cardiovascular protective, cytoprotective, neuroprotective, and immunoregulatory effects (121); Treatment of high blood glucose and lipid levels and other symptoms of the metabolic syndrome, polycystic ovary syndrome (PCOS) and inflammatory disorders (122).	Occasional gastrointestinal disorders and allergic reactions reported (121); Potential nephrotoxicity and hepatotoxicity at higher than recommended daily dose (123).	Trans-cinnamaldehyde: USCFR GRAS, § 182.60.
Vanillin, ethyl vanillin, vanillic acid	Antioxidant, anti-inflammatory, antisickling, antimicrobial, and hypolipidemic effects; prevention of cancer, periodontal disease, and bone deterioration (124)	Lack of toxicity at approved levels of intake in foods; Vanillin may induce bronchoconstriction in asthmatics (125), contact dermatitis at high concentrations (126).	Vanilla extracts: USCFR GRAS, §182.20; Vanillin and ethyl vanillin: USCFR GRAS §182.60 (Synthetic flavoring substances and adjuvants, can be from natural sources); Vanillic acid is not listed in the US FDA Code of Federal Regulations; evaluation by the FAO/WHO Expert Committee on Food Additives (JECFA no. 959) yielded “no safety concern at current levels of intake when used as a flavoring agent” (127).
Caprylic acid	Role in the prevention of infection and inflammation as part of lipid emulsions used in parenteral feeding of neonates (128); Prevention of obesity by decreasing energy intake, possible effects on appetite (129).	No evidence of toxic effects at doses up to 10% in the diet (130).	USCFR GRAS §184.1025; Available as a dietary supplement.
Thymoquinone	Anti-inflammatory, antimicrobial, antiparasitic, antioxidant, antihyperglycemic, and anticancer properties (131).	Concentration dependant *in vitro* hepato-toxic effects (132); No evidence of cytotoxicity in rats (133); no evidence of toxicity in humans at daily oral doses up to 28 g/kg (134).	Source plant (*Nigella sativa* L., black seed or black cumin), is listed by USCFR GRAS in § 182.10 (Spices and other natural seasonings and flavorings); Source plant extracts listed as dietary supplements under the Health and Education Act of 1994 (120).
Coenzyme Q_0_	Antitumor, anti-inflammatory and anti-angiogenic effects (82).	No evidence of toxicological effects from dietary supplements (135).	Not presently permitted as a food additive. Available as a dietary supplement.
Nisin	Prevention of dental caries (136); anticancer and antibacterial (137).	Effects on the cytoskeleton of keratinocytes derived from normal epithelium; increased blood cholesterol concentrations in rats (138).	USCFR GRAS, §184.1538, antimicrobial for specified uses which do not currently include PIF.
Lactoperoxidase	Inactivation of carcinogens (139); Contributions to cytotoxic effects against human cancer cells (140); Prevention of bone resorption through osteoclastogenesis (141).	Preparations derived from bovine milk could contain proteins which may be allergenic for sensitive individuals.	USCFR GRAS notice granted for lactoperoxidase system as a processing aid for dairy products pursuant to § 170.30 (Eligibility for classification as generally recognized as safe (GRAS).
Bovine lactoferrin	Contributions to cytotoxic effects against human cancer cells (139, 142); Contribution to gut health and immune development in neonates (143, 144); Prevention of acute gastrointestinal and respiratory symptoms in children aged 12–32 months (145).	No adverse effects in rats fed 2,000 mg/kg/day bovine lactoferrin for 13 days (146); Considered safe for human consumption (147).	USCFR GRAS notice granted for cow's milk-derived lactoferrin as an additive ingredient for PIF pursuant to §170.35 (Affirmation of generally recognized as safe (GRAS) status).
Cell free extracts of *Lactobacillus* spp.	Variable, depending on species and nature of extracts; Management of intestinal, respiratory diseases (148); Cytotoxic effects against human cancer cells (149); Immunomodulation, anti-inflammatory, antiproliferative, hepatoprotective effects (97).	No evidence of adverse effects from oral use.	USCFR GRAS notices have been granted for some cell free extracts; Several are available as a dietary supplements.

All NACs from plant sources with antibacterial activity in PIF listed in Table 4, with the exception of caprylic acid, are either purified phenolic compounds or contain high concentrations thereof. Vanillin, ethyl vanillin, and trans-cinnamaldehyde have GRAS status, and are widely consumed in food and nutritional supplements. Several vanilla-flavored PIF products containing natural and artificial flavors were once available in the marketplace but have since been withdrawn, although vanilla flavored “toddler” formula is still sold in North America. Whether the level of vanillin or ethyl vanillin supplementation used in these products was sufficient to achieve antibacterial effects against *Cronobacter* during PIF reconstitution or storage, as reported by Polat Yemiş et al. (72) is unknown. Supplementation of PIF with cocoa powder was also discontinued several years ago. The antibacterial activity of cocoa powder against *C. sakzakii* in reconstituted PIF described by Pina-Pérez et al. (58) was likely derived from polyphenols, many of which are known to interact with and damage the bacterial cell membrane (83). Polyphenols encompass several groups of compounds including phenolic acids and flavonoids, primarily flavanols present as monomeric epicatechin, catechin, and their oligomers referred to as proanthocyanidins. Research on the nutritional effects of moderate cocoa consumption suggests that the benefits likely outweigh the risks, and that beneficial effects on health are primarily derived from flavanol-mediated protection against oxidative insult by the modulation of oxygen radical generation and antioxidant enzyme and non-enzyme defenses (151, 152). There is compelling evidence that most polyphenols are largely beneficial to human health, principally for the prevention and management of chronic diseases (153). On the other hand, the pharmacological properties of some polyphenols introduce concerns about their safety in products intended for use by infants. Isoflavones (genistein, daidzein, and glycitein) derived from soya beans are known activators of estrogen receptors with demonstrable effects on reproductive and endocrine functions in animal models (154). All soy-protein based PIFs contain isoflavones, mainly genistein, but no clear consensus has emerged regarding the short or long term implications of long-term dietary exposure on the development of infants (155, 156). In contrast, consumption of the medium length straight chain fatty acid caprylic acid (octanoic acid) is considered to be comparatively free of toxicological risk (130). Caprylic acid is found naturally in the milk of mammals including humans (157) and in infant formulas as part of the medium chain triglyceride component contributed by vegetable fat, or increasingly bovine milk fat (158). Choi et al. (74) showed that low concentrations of caprylic acid in conjunction with citric acid completely inactivated *C. sakazakii* in PIF during reconstitution at the relatively low temperature of 45°C. A GRAS compound, it is used as an additive in a range of foods as an adjuvant or, interestingly, as a flavoring agent despite having an odor described as “slightly unpleasant and rancid-like.” Similarly, the monoterpene diketone thymoquinone, also a GRAS compound, could inactivate *C. sakazakii* during reconstitution (78) but it has a bitter taste and a “pencil-like” odor (159). The ubiquinone coenzyme Q_0_ is the only ordorless and tasteless NAC from non-microbial or animal sources identified to date with antibacterial activity against *Cronobacter* spp. Coenzyme Q_0_ extracted from the AC mushroom (*Antrodia cinnamomea)*, a parasitic fungus that grows of the camphor tree, has a long history of use in traditional medicine but has only recently been considered for food applications. Toxicological assessment of supplements prepared from the fungus suggest they are safe for human consumption (132). However, toxicological assessments of the purified compound are lacking and coenzyme Q_0_ does not currently have GRAS status.

The chemistry, biology, toxicology, pharmacokinetic properties, and functionality of nisin as a food preservative have been extensively investigated. A recent reassessment of toxicological data by the European Food Safety Authority (EFSA) reaffirmed the safety of nisin as a food additive (160). Activity against *Cronobacter* spp. in PIF relies on synergism with citric acid (92), but the latter is a permitted additive. Hence, there appear to be few regulatory impediments to the use of nisin in foods destined for infants. As noted above, antibacterial activity of lactoperoxidase, another GRAS food additive that is used worldwide for milk preservation, is dependent on a source of thiocyanate, which Gurtler and Beuchat (101) provided exogenously in the form of sodium thiocyanate. Thiocyanates are ubiquitous in food products, however, and it is unfortunate that no attempt was made to determine if endogenous levels could have sustained the reaction. Evidence in support of this presumption was provided by Banks and Board (161) who found that lactoperoxidase catalyzed degradation of endogenous thiocyanates reduced the growth of *Enterococcus, Pseudomonas* spp. and Enterobacteriaceae in reconstituted PIF stored at 30°C for 48 h, which coincided with the depletion of free SCN- ions. These observations suggest that the value of lactoperoxidase for the control of *Cronobacter* spp. in PIF merits further investigation. There are also few regulatory obstacles to the application of lactoferrin in PIF since it is already in use for therapeutic purposes, disease prevention or health promotion in neonates (82, 143, 162), and is available in highly purified forms safe for use in infant foods (163). Cell-free extracts derived from microbial cultures present greater regulatory challenges as noted above due to the multiplicity of bioactive compounds and variable composition of extracts which add complexity to toxicological assessments. In this context, the selection of candidate microorganisms for the production of cell free-extracts among those already permitted as probiotics in PIF or that are considered GRAS on the basis of historical, safe use in food fermentations is highly advisable.

## Conclusions and Future Prospects

Societal concerns and regulatory response to the risk of exposure to harmful food chemicals in early life provide strong impetus to pursue the search for alternatives. Research on NACs with antibacterial activity against *Cronobacter* spp. has shown that several could find value in the control of this hazardous pathogen in PIF. However, technological obstacles to practical applications persist and means to overcome them must be the focus of future research in the field. The delivery of NACs with strong antibacterial activity to food systems is often hampered by limited solubility in aqueous matrices, instability, reactions with other food components or adverse effects on sensory properties. For example, the low solubility, volatility, intense sensory characteristics, and reactivity of phenolic compounds and essential oils often hinders their incorporation in foods. Adverse effects on the sensory quality of PIF are a notable concern in light of evidence that exposure to flavors modulates neonatal feeding behavior and food acceptability and choice later in life (164, 165). Recent progress in the use of biopolymers from natural sources for the design of innovative encapsulation systems that provide means to deliver effective yet reduced doses of antibacterial agents, protect active ingredients from undesirable reactions, and provide controlled, quantitative release into food matrices will undoubtedly promote the development of delivery strategies that overcome constraints on both the choice and application of NACs (166, 167). The selection of suitable NACs must also be guided by careful consideration of the regulatory framework governing PIF composition, specifically the use of additives. In the near term, single compounds for which nutritional and toxicological data are available should take preference over preparations likely to contain multiple bioactive compounds. The latter remain eminently worthy of study, however, as possible sources of novel NACs. As a final note, it must be emphasized that the NACs described in the present work were derived from a limited number of animal, plant and bacterial species. Future efforts should be directed at the assessment of additional sources of NACs for the control of *Cronobacter* spp., such as mushroom species known to contain compounds with antibacterial activity against other foodborne pathogens (168).

## Author Contributions

GP and PD equally contributed to a review of the scientific literature, collection of relevant references, writing, and editing of the manuscript. All authors contributed to the article and approved the submitted version.

## Conflict of Interest

The authors declare that the research was conducted in the absence of any commercial or financial relationships that could be construed as a potential conflict of interest.
